# Cannabis consumer responses to the concept of standard THC units

**DOI:** 10.1111/add.70515

**Published:** 2026-06-22

**Authors:** Mark Pawson, Mike Vuolo, Brian C. Kelly

**Affiliations:** ^1^ Department of Sociology Purdue University West Lafayette IN USA; ^2^ Department of Sociology The Ohio State University Columbus OH USA; ^3^ Department of Sociology Indiana University Bloomington IN USA

**Keywords:** cannabis, standard THC unit, risk management, harm reduction, consumers, risk perception

## Abstract

**Aims:**

Within a context of considerable cannabis market expansion and product diversification, cannabis consumers must manage their use across a range of potencies and modes of consumption. This study aimed to provide deeper understandings of how cannabis consumers would perceive the introduction of a standard THC (tetrahydrocannabinol) unit system.

**Design:**

Qualitative study.

**Setting:**

United States, 2023–2024.

**Participants:**

Adult cannabis consumers (n = 50) across criminalized and legalized cannabis states.

**Measurements:**

Within a study focused on the experiences of dosing cannabis among adults in contexts of evolving cannabis markets with product diversification, subjects were provided with a description of a standard THC unit and with the analog of a standard drink. They were then asked about their perceptions of a standard THC unit. Interviewers probed for personal attitudes, practical utility and potential for reducing harm.

**Findings:**

Broadly, cannabis consumers were enthusiastic about the introduction of a standard THC unit system, although a minority expressed some skepticism about how it would be implemented. Standard THC units were viewed as most beneficial for novices and infrequent consumers, although some experienced consumers indicated it would be valuable for tolerance management and avoiding dependence. It was also seen as valuable for avoiding risks related to driving under the influence and overconsumption.

**Conclusions:**

Standard metrics for cannabis consumption may be received with enthusiasm from cannabis consumers. Experienced consumers indicated they would have benefited from a standard THC (tetrahydrocannabinol) unit when initiating cannabis use and highly recommended it for novices. It was also viewed as an opportunity to manage risks. Successful implementation would require managing misunderstandings about the concept of a standard THC unit as well as how regular, experienced consumers would incorporate such a metric into established consumption routines.

## INTRODUCTION

Legalization of cannabis in several countries has enabled vast diversification of available cannabis products with a wider range of potencies [1, 2]. These policy driven changes and restructuring of market forces represent a shift in the risk environment, reshaping potential vulnerabilities related to cannabis consumption [3]. Consumers have more numerous product options than in the past. Beyond cannabis flower, cannabis dispensaries (and gray market sales extending from them) sell vaporizers, edibles, concentrates, tinctures and drinks, among other product types. In addition to diversification of products, potency has increased over time as the cannabis market evolved. Even within product types, potency of Δ‐9‐tetrahydrocannabinol (THC) content can vary greatly [4], which can lead to uncertainty about how to consume different cannabis products in ways that maximize desired outcomes and reduce its risks. Therefore, diversification of legally available cannabis products, and large variations in their potency, complicates consumers' efforts to consistently use cannabis across a range of products to produce reliable effects and manage associated risks [5].

Although legal cannabis companies often provide product potency information previously unavailable through illicit markets, this information is not always consistent across product types. For instance, although some United States (US) states require companies to provide such information on cannabis products, not all government bodies set a universal standard across jurisdictions. The lack of a consistent system across locations and displayed in ways that are clear and obvious to consumers complicates efforts to establish socially embedded norms for acceptable (versus potentially problematic) consumption dosages. Because of variability between product types as well as variation of potency within product types, there is a great need to identify how individuals understand cannabis consumption across distinct product types within a context of increasing product diversification. Although metrics of standardization have been proposed to assist consumers [6, 7], there is a need to understand how individuals comprehend and would use standard cannabis ‘units’ in their consumption experiences.

Concerns about reducing risks associated with cannabis use have become more prominent as consumption expands [8]. The concept of standard cannabis units provides opportunities for consumers to intervene on the harms of cannabis use, both acute and long‐term [6, 7]. Modeled after the measurement of a ‘standard drink’ for alcohol use [9, 10], calls have been made for an internationally recognized standardized measurement of cannabis consumption [11, 12]. The establishment of a standard drink and accessible measurements of problem alcohol use, such as binge drinking, not only more easily enables identification of problematic patterns of consumption, but also allows consumers opportunities to self‐regulate consumption, regardless of beverage choice [13, 14]. With cannabis, this has become especially important given the growing diversity of product types.

The US National Institute on Drug Abuse (NIDA) recognized 5 mg of THC as a standard unit for research [15]. Although cannabis products may contain potency information, and research demonstrates that consumers use this information to make decisions about consumption [16], no consistent method of delivering this information is used to clarify dosage across product types (e.g. 25% THC cannabis flower versus 80% THC vaping pen) or between potencies within product type (e.g. 20% THC cannabis flower versus 30% cannabis THC flower). Furthermore, consumer confusion might ensue with different metrics, with cannabis flower typically marketed by % THC and other products by mg THC (e.g. edibles) or even both (e.g. vaping devices). Nonetheless, advocacy for a standard cannabis dosing metric—often referred to as a ‘standard THC unit’ or ‘standard joint unit’—favors providing consumers with a more consistent sense of their consumption, regardless of product type or potency [6, 7]. Such information may expand opportunities to improve public health, not only by providing clinicians and public health professionals benchmarks from which to reduce problem consumption and promote health, but also by providing consumers with consistent information to make well‐informed decisions to mitigate risks.

With increasing professional calls for standardization of cannabis potency information within a shifting risk environment, a need to understand how consumers would perceive the introduction of a standard THC unit system exists. Identifying explanatory models from consumers is vital for proper implementation. As stated about substance use by Agar, ‘a variety of interpretations of the same object are possible, depending on the actors who encounter it and the traditions in which they participate’. [17] This is especially important given that health professionals and consumers often have different working understandings of risk and prevention efforts [18]. Professional models typically occupy a privileged status in a society; they are imbued with authority and offer official interpretations of a given behavior [16]. In contrast, ‘folk models’ arise popularly through the daily practices of people, and they align with the lived experiences of the population of interest. Nonetheless, these understandings are essential for the implementation of procedures to promote health and reduce risks [19]. Active consumers are an important group to target given that they are more likely to adopt new innovations in technologies and procedures [20].

### Current study

We aim to characterize how adult regular cannabis consumers perceive and understand standard THC units. Within an interview study about dosing across different cannabis products in a changing risk environment, we queried consumers about their reactions to the concept of a standardized metric to track cannabis consumption. The concept of a standard THC unit was presented to them and subjects were asked for their opinions in open‐ended fashion. The following results provide a thematic analysis of how adult consumers perceive the concept of standard THC units.

## METHODS

### Design and sample

The data come from a qualitative in‐depth interview study focused on experiences of dosing cannabis within contexts of evolving cannabis markets with product diversification. Our interview sample consists of 50 US adults who actively use cannabis. Inclusion criteria specified cannabis use at least monthly and experience with products from legal cannabis marketplaces, and there were no restrictions on types of cannabis products used. Patterns of consumption varied across the sample, with some consumers using daily while others used cannabis less frequently. Notably, participants lived across different state policy contexts, spanning legal recreational, legal medical and criminalized contexts. Participants must be US residents, although not restricted on state of residence. We note that even in criminalized states, most consumers reported purchasing cannabis within legal environments. The sample was recruited via flyers posted around two US Midwest cities (one in a state prohibiting cannabis and another with legalized recreational cannabis) as well as at cannabis dispensaries in two other legal recreational states. Direct engagement with potential subjects during fieldwork at dispensaries as well as snowball sampling supplemented flyer‐based recruitment. Consumption frequency ranged from monthly to daily. Consumers typically had experience across multiple cannabis product types. Most participants were exclusively recreational consumers (i.e. no medical use). As shown in Table 1, the sample consisted of 22 men and 28 women. Aligned with age patterns of cannabis consumption, the vast majority (92%) were young adults under age 40. Approximately two‐thirds of the sample was white. The 50 participants resided across 11 states: 17 in states with legally available cannabis and 33 in states prohibiting cannabis (most living in criminalized cannabis states adjacent to states with legal cannabis markets).

**TABLE 1:** Sample characteristics.

	*n*	%
Gender		
Male	22	44
Female	28	56
Age, years		
20s	37	74
30s	9	18
40s	2	4
50s	2	4
Race		
White	34	68
Non‐White	16	32
State policy context		
Recreational/medical	17	34
Prohibition	33	66

### In‐depth interview procedures

All interviews were traditional individual in‐depth interviews conducted during 2023 and 2024. Interviews were primarily conducted by the first author, and the third author assisted with the interviews. The semi‐structured interview guide covered a range of issues related to how subjects manage dosing and consumption across a range of cannabis products. Within the guide, subjects were asked ‘Tell me what you know about a standard THC unit or a standard cannabis unit?’ It was anticipated many would be unfamiliar and subjects were then given a description of a standard THC unit and how it compared to the concept of a standard drink (please see Appendix S1 for a description). Participants were then asked to respond to questions about the concept of a standard THC unit and whether they considered it potentially useful for cannabis consumers, such as ‘How could this type of information be useful to you when you use cannabis?’ Questions about standard THC units were phrased in an open‐ended fashion to receive subjects' opinions in a broad manner amenable to a grounded analysis of consumer perspectives. The interviewers used probes to draw out details from participants to gain greater precision and deeper explanations of their perspectives. All interviews were digitally audio‐recorded and transcribed verbatim.

### Analytic approach

We analyzed data through an iterative process. Interviews were coded initially using a combination of inductive and deductive perspectives [21]. We approached the data through a process of multiple readings, coding and assessing emerging concepts and themes for relevant mechanisms [22]. The initial wave of coding was completed by the third author, followed by an additional wave of coding by the first author. At each point, all codes and their application were discussed by all three authors to achieve consensus before organization of the themes yielded from the analysis. Inductively, multiple readings of the data allowed for the discovery of emerging themes to permit contrasting experiences to possible mechanisms. Yet, as argued by Strauss, we also applied a deductive approach, making theoretically sensitive coding an important component, keeping in view the goal of our analysis [21]. A specific goal was to identify how standard THC units resonated with the experiences of consumers. In this respect, we evaluated how adults described their perspectives on standard units and how these related to their cannabis use.

During this iterative process, subsequent application of codes in consideration of theoretical frameworks ultimately cohered with that of a thematic analysis, a qualitative analytic technique used to identify and analyze patterns in descriptions of particular phenomena [23]. Our approach aligned with standard qualitative analytic techniques to enable the distillation of wider themes from the narratives provided by subjects, while leaving outlying individual assertions aside. We also sought analytic contrasts in potential subject disagreement. Consequently, reported results reflect themes endorsed by multiple respondents, and these themes are exemplified with direct quotes for illustration. Quotations are indicated by user ID, and we aimed to use quotations from a range of subjects to represent themes (quotations below were yielded from approximately half the sample). For reference, Table 2 contains a list of themes and subthemes generated from this analysis. For this study, NVIVO and Excel were used for management of codes and analysis of data. The study received institutional review board approval (#2022–1081) and consent was obtained from subjects.

**TABLE 2:** Analytic themes.

Primary theme	Sub‐themes
Medical use	Medical value; dosing concerns; medical labelling
Precision	Benefits of exact dose; trial and error; reduce confusion; guidelines
Problem assessment	Tracking consumption (short‐term); tracking consumption (long‐term); tolerance management
Utility for novices	Social learning; needed for inexperienced; less value for experienced
Comparisons to drinking	Familiarity of comparison across drinks; educational benefit
Preventing harm	Overconsumption; greening out; dependence; bad experiences
Remaining uncertainty	Skepticism of utility; challenges with flower; different effects across consumers; government restrictions

## RESULTS

When the concept of standard THC units was described, most consumers indicated they had not previously heard of a standard metric for tracking consumption across diverse product types. Yet, most were very receptive to the idea of standard THC units across products when it was described to them, saying things such as ‘I think that would be very helpful.’ (244), ‘I'd definitely say yeah.’ (228) and ‘For me, absolutely.’ (260). Within interviews, subjects articulated a detailed range of advantages to this information (described below) and indicated they wanted to see a standard system that provided clearer information about potency across different products. Many stated they wanted this information visibly identified on packaging and within dispensaries. Although not uniform in their perspectives on standard THC units, generally, most subjects expressed strongly positive views on standard THC units.

### Desire for greater precision

A common issue raised by consumers was their desire for greater precision and a clearer basis for comparisons to inform how they make decisions about consumption across different cannabis product types. Especially for those who use different products and potencies, they indicated it could potentially reduce confusion.

‘I think I would strongly be inclined towards it, if there's a unit for THC…it helps me in not doing all these calculations. Just like go into a store and picking up the same how many units of THC. So, I think yeah. Yeah, that's definitely a thumbs up from my side. That's a great idea.’ (207)

‘Yeah, a lot of times it's very confusing. You just see milligrams or even like percent THC and percent CBD, all these percent numbers. And I have to study on my cell phone like what that means so I can make a decision. But yeah, at first that was all very confusing.’ (206)

‘I'm a numbers girl, so numbers just make sense to me. So, if I were able to put like a unit on it, like that would make it so much easier. It would. It would be nice for me, yeah.’ (253)

It is notable that several consumers described explicitly trying to calculate consumption. There is already an intentionality to understanding distinctions in potency across products. This finding indicates that, rather than haphazardly purchasing cannabis, many consumers aim to determine in advance of consumption their expectations across different product options, and they want tools to facilitate this and reduce the pressure of figuring it out on their own.

Interviewees also regularly described a process of trial and error to learn how much cannabis was appropriate for them in different circumstances. They emphasized how a standard THC unit would have made that process easier and with less risk.

‘I think if you were to take me back like 2 years to when I first started using semi‐regularly…it probably would have taken out that learning curve. I probably would’ve been like this is enough, this is too much, because I’ve had enough experiences with having too little or having too much that I know what's too little, what's too much. So, I think that would have been really, really helpful actually.’ (205)

The information was also seen as valuable for tracking and managing consumption. Several interviewees described that it would help them track their use over the course of an evening or even over the course of a more extended time period. This was seen as especially valuable for reducing the amount of trial and error.

‘It would give me an easier way to track how much I'm smoking as opposed to just kind of the methods that I've come up with by myself.’ (236)

‘Yeah, absolutely. I feel the more information the better. At least, you kind of have a guide even if you use it as a jumping off point if this is the standard; this is what typically it is. Or even across different things, I don't typically use edibles. I haven't used one in so long that if I were to start again, figuring out what the right dose is for me would be more difficult. So, having 5 mg of this is compared to this? I'd be OK, I can better gauge.’ (212)

We also note that although most participants were exclusively recreational consumers, they nonetheless identified the utility of standard units for medical use. Although not discussed extensively here, these responses aligned with a desire for greater precision, as standardization could assist consistent medical use.

### Comparison to drinking useful for monitoring consumption

Several consumers felt comparisons to standard drinks were intuitive. They suggested it would allow them to monitor consumption over the course of an evening in ways similar to how people track drinks. In this manner, a standard metric for cannabis based upon the concept of a standard drink seemed to resonate for many interviewees. Importantly, it resonated with consumers even with different perspectives on the sufficiency of 5 mg as a standard unit.

‘It feels like a good amount. It does because it's enough for newer smokers to definitely feel it and know. And even if you've smoked in the past, you're still going to feel 5 mg. But I know people tend to go a lot higher than that, simply like similarly to how they do with alcohol, where a lot of times people don't just have one drink.’ (236)

‘Well 5 mg is low so let's see…when you're at a bar you don't have just one drink. So, you know, nobody is using just like a single milligram or even 5 mg. But yeah, like you know you usually have like 5 or 6. You know, maybe if you're just having one drink, in that case the gummies are 5 mg so yeah I guess just one would be fine, but it's not intended to be like for the night.’ (206)

Comparisons to drinking were also made by interviewees for the sake of reducing problems, such as driving under the influence, for which legal and social standards are more well established for alcohol. In this manner, they viewed standard THC units as a means for reducing some of the ancillary problems associated with cannabis use

‘If I hear that someone had X number of drinks, I know what that means and that is useful for understanding information. I mean, I do think about stuff like, I don't feel myself impaired to drive, but like that is a really valid question that people have about legalization. How do we sort of quantify what is OK and not OK when we're talking about other people's safety?’ (222)

‘Definitely. I feel like if you think of BAC, blood alcohol content, right. You know, you can just feel like, oh, I've had one too many drinks, so therefore, I can't drive, right?… The thing is like, there's a certain amount that you can consume where you're like you're still functioning and you're still fine. Of course, no you should not drive high, I'm not condoning that at all. Don't drive under the influence at all. Like you have like a beer and drive. You know, you're not like, Oh my God, like on the floor or something like that, right? We have blood alcohol content.’ (253)

The concept of standard units was also seen by some as a potential health education tool to deploy in more formalized settings.

‘Well…one shot is about one glass of wine, which is about a beer. You have these scales that are commonly taught, even if they’re not taught in a way of “this is how you should drink”, but just like this is what you can expect. If something like the 5 mg is brought up, where it's like 5 mg of an edible is equivalent to like this equivalent of smoked flower or this amount of resin smoked…things like that. I just feel like if there's a little infograph that you could get in school just like a slide that they could cover in health class. I feel like that would have a somewhat lasting effect.’ (227)

### Utility for novices and infrequent consumers

Although responses to the concept of standard THC units were largely favorable, a notable proportion emphasized that the introduction of standard units was most beneficial for new consumers or those who infrequently used cannabis. Accordingly, infrequent consumers or the newly initiated were seen as the primary target group for implementation of a standard metric.

‘I think it would be helpful for people who like haven't smoked a lot or don't know a lot about like their tolerance personally. The first time I ever smoked, I had people who were avid smokers so they all knew what they were doing so they kind of taught me what to do. But I guess if you don't have that (group of people), it would be nice to have that.’ (202)

‘It's interesting. I think that's a good standard dose [5 mg] because from my experience with friends who are new to smoking or just wanting to try it, that seems to be their limit, especially with edibles some will even have half of it. So I think that's really smart for people starting out. It's a good guideline for sure.’ (233)

Yet, although some consumers highlighted utility for novices or infrequent consumers, they used it as an opportunity to distance themselves from the use of a standard unit. In doing so, these consumers positioned themselves as experts who did not need a standard metric and, therefore, diminished the utility of standard units.

‘I don't think it make[s] any difference for me, but I mean for new smokers and new users of marijuana or cannabis, I think it would be pretty helpful to see, you know, this is standard size.’ (248)

‘I think it's like probably better to say it's just for people who don't use it as often. And they're the ones who kind of need the guidance, I think, more than a veteran smoker, you know? Yeah, but I don't know. I think again, I don't know what a better solution would be.’ (267)

The role of perceptions of expertise and the role of routines in consumption may have an effect on the willingness of some experienced cannabis consumers to use a standard metric.

### Preventing harm and problem assessment

Subjects identified reducing potential problems as a great benefit of standard THC units. Many interviewees quickly pointed to the prevention of overconsumption. As such, not only would standard THC units permit the management of bad health outcomes, but also facilitate more pleasant consumer experiences.

‘I feel like some people, you know, accidentally ingest way more or smoke way more than they would really want to, just because they don't know how to measure it. So yeah, I think that would be something that would be really beneficial to kind of prevent people from going past their personal limit.’ (263)

‘Yeah, that is really helpful actually. I mean, I figure, it's like obviously it's easier to measure drinks, but I think it is helpful because it helps people to not green out or not do too much, so I think there should be a good standard measurement for that.’ (211) (Note: ‘greening out’ is a term used by consumers to refer to nausea/vomiting resulting from the overconsumption of cannabis)

‘I for sure think that 5 [mg] is better than 10 [mg] because, to be honest, because a lot of times, I tell people to start with half. I think that's a better starting point. As a consumer, when you see a gummy, especially from a dispensary, you're going to eat the whole gummy, right? And I think being able to have a lower dosage and enable people to build up instead of just smacking you in the face with a 10, because a 10 can do nothing to some people. Meanwhile, a 10 for me is like a nice party time. It can be different for so many different people, that I think having a lower standard dose would be the safer side of things, where people are having better experiences because they're not overdoing it.’ (218)

In addition to managing acute problems from overconsumption, others felt it would be useful to gauge consumption to monitor longer term issues. These issues specifically included both prevention of dependence as well as the management of tolerance.

‘Relating it to drinking, it's like harder to tell because I feel like it's not a problem. It's not like dependency or an addiction or something. It's not that big of a deal, right? Because with drinking, if you are constantly drinking this much or like you're going every single day…that's a problem. And I think it's the same thing for weed. If you think that you have a problem with it, maybe refer back to that dosage to kind of wean yourself back off of it.’ (202)

‘I mean you know how to see the dosage and you don't want to take too much. And again, it's not lethal but it is uncomfortable. Again, I think one of the things I missed out was guidance on how often you should smoke. A lot of my friends just sort of wake up and smoke and they just go about their days. But that's the reason I only smoke like once or twice a week. It keeps my tolerance down and I worry about those friends, and I use them as an example of don't do it like that. But you don't really have any guidance on what's a normal amount to use, as they just say don't use any at all.’ (206)

‘I think if I were wanting to, for example, cut back or to like get my tolerance up or something like that, it would be easier to measure. Okay, I'm cutting back this much, and then I'm going back up this much. So as far as being able to control my tolerance, a dosage would be useful.’ (203)

These issues were of concern to the more regular consumers of cannabis, such as those who used cannabis daily or near daily, as well as those who consumed less frequently.

### Remaining uncertainties

Although most interviewees were highly positive about standard THC units, a minority expressed skepticism about how it would be implemented and whether it would ultimately be beneficial for most consumers. A particular concern expressed was the variability in the effects of cannabis on consumers, because of individual size differences as well as differences in tolerance over time.

‘I don't think so, because it affects everyone drastically very different. I've had friends that, you know, if I have some 25 mg edibles I've given to other people, they've taken one and they've been blasted. For me, I can take three and be fine. So, it is very different for everyone. I can understand 5 [mg] would be a safe bet, but it seems just very, very low to me. I think more research needs to be done about your body weight, how that affects the dosage. Same thing with alcohol, like how much food have you had in your system. Things like that I think is really important to play in.’ (252)

‘I just feel like with each individual there's a limit. Some people are very high tolerance, some people have a low tolerance. As I said, for me, I can't smoke a whole blunt in a sitting, right? But some people can. And they'll get as high as I got when I smoked that smaller amount. I don't know. I don't think it would be something that…well, it'll probably make a slight change, but it wouldn't make a big change. Also once again, it goes to your tolerance. I remember when I first started smoking, I would freak out because I didn't really understand the concept. But now that I smoke, I know that, you know? I know the effects of it. I don't even like saying this, but I can operate really well compared to the first time I started smoking.’ (266)

Others' skepticism focused on concerns that standard units would be more useful for gummies and other edibles, but would be impractical for individuals smoking cannabis flower.

‘I feel that the milligrams would only make sense if someone was having an edible, because you can't really measure that dosage when you're smoking flower or even concentrates because you only get how much percentage is inside of it, but you don't really get how much your dosage is. Even when you are eating edibles from the black market, they usually tell you I have no idea. I'm just eating half of that bar and so measuring an edible, I can understand the sense of it. OK, this piece is this much dosage, But if you like, if you gave someone a pre‐roll, you can't say this is 5 mg of weed right now. Or like THC. There's no way of like, really regulating that dosage. Because it comes in different forms.’ (219)

‘It could be helpful, but I feel like it's really hard to do that. You're probably right. You're gonna be like milligrams if they do one. But a lot of people just don't do it that way. They just do a joint. And there's like different sized joints, there's small, big, huge. So how are you say, oh, one joint is good for a day and this person's joint is like this big? That's not gonna work out.’ (224)

‘I feel like it would be hard to control because every single strain has a different percentage and so like, you can't just be like one bowl because one bowl is gonna have varying amounts of chemicals. Even, if you pull out a nug, that nug could be different than the next one you pull out, so you never even know.’ (257)

As suggested above, some of the skepticism may also be because of misunderstandings about how standard units would be applied across products, including cannabis flower. Others questioned its use given that people have differing tolerances. Similar issues arise with alcohol, as standard drinks must be managed with differing alcohol by volume both within and between product categories. This raises questions about the ease with which standard THC units may be rolled out without prior research and accompanying educational initiatives.

## DISCUSSION

Addiction scientists and public health professionals have introduced the concept of standardized cannabis units to intervene on potential acute and long‐term risks of cannabis use and to make the experience of cannabis use more reliably pleasant [7, 8]. We aimed to understand consumers' explanatory models of such a metric [17, 18]. Overall, we found considerable enthusiasm for standard THC units among adult cannabis consumers in the United States. This is especially important within a context of several interviewees volunteering that they intentionally attempt to calculate doses across products at the time of purchase and that having a standard metric would be valuable in such instances. Such intentionality primes consumers for the availability of a standard metric.

Although almost none of the interviewees had heard of the concept of standard units previously, most found potential implementation useful for managing consumption. Importantly, even without prior knowledge about a standard metric, subjects identified many potential benefits of standard THC units recommended by health professionals (e.g. López‐Pelayo *et al*. [7] and Lorenzetti *et al*. [12]). As the proportion of the market occupied by cannabis flower has declined with alternative products (e.g. vaping devices, concentrates and edibles) becoming increasingly popular and more widely available [24], such product diversification is especially important within a background of increasing potency [25]. Within this context, cannabis consumers' conceptions of standard THC units largely align with those intended by professionals, which enhances the potential for successful implementation for the purpose of harm reduction [19]. Clear information on the product is essential for a well‐regulated market [26], and consumers recognize and desire to manage consumption within this increasing range of options. Other research has identified a desire for improved health warning labels on cannabis packaging [27], and the provision of a standard THC unit marked on products in a clear and unambiguous manner may also enhance communication of such information to consumers. In this context, cannabis consumers' desire for greater precision begins within the marketplace, where standard units could be useful for making decisions about purchases, and extended to the act of consumption, where standard units were viewed as desirable for titrating doses.

Many consumers pointed specifically to reducing the probability of acute problems from overconsumption, such as the risk of ‘greening out’. Managing consumption during a session of use was a key concern identified across many interviewees. In this regard, they directly identified potential harm reduction benefits with the implementation of a standard metric. Yet, some also moved beyond concerns for immediate problems with acute intoxication to identify how a standard metric could assist with handling concerns about dependence and managing tolerance. Indeed, some scholars have argued that a standard metric for cannabis could enable clearer markers of high‐risk cannabis use [28]. Aligned with this, consumers expressed that they wanted a more structured means to aid their efforts to prevent dependence. Tolerance management was also a key concern, not simply because of the risk of dependence, but also because of rising costs of increasing consumption. Standard THC units were seen as a useful means to track consumption to manage tolerance. Interviewees also identified its potential utility for managing concerns about driving while intoxicated, which has been recently identified as a means to facilitate safe driving within legalized contexts [29].

Although standard THC units were widely seen by interviewees as most beneficial to novice or infrequent consumers, there may be downsides to such assertions. Many consumers interviewed were well experienced with cannabis, and while many were enthusiastic, some professed no personal value for a standard metric given their perceived expertise. Perceived expertise can lead individuals to eschew new alternatives—the earned dogmatism effect—and in turn reduces uptake of potentially beneficial innovations [30]. Subjective knowledge is associated with self‐limiting options in decision making, and such knowledge can have negative effects for consumers more broadly [31]. This finding aligns with other research indicating that frequent consumers are less sensitive to health messaging on cannabis packaging [32]. It is also situated within a wider culture of increasing skepticism of health guidelines and medical recommendations more generally [33]. Furthermore, research on standard drinks suggests that among those indicating knowledge of a standard drink, fewer than half accurately estimate it in practice, which can lead to overconsumption and challenges with problem identification [34]. Therefore, perceived expertise among experienced consumers may be a barrier to wider adoption of standard THC units for the purpose of harm reduction. Yet, as identified by several interviewees, standard THC units hold promise for managing consumption to prevent the development of dependence as well as for managing tolerance in a more systematic way. Should these opportunities be dismissed by experienced consumers the potential for intervening on tolerance and dependence will be reduced, and therefore, this is a focal point for intervention.

Among the minority of consumers who identified uncertainties about how useful standard THC units would be in practice, skepticism centered on personal utility and implementation across different products and tolerance levels. There have been similar challenges associated with the introduction of standard drinks [35]. Yet, some of their stated uncertainties raise concerns about how well some consumers understand the concept of a standard metric, even after having it explained to them. Despite that, standard THC units' value lies with the ability to make clearer comparisons across products, some consumers did not see applicability to cannabis flower or other modalities assessed via % THC. The perception of utility for some products, but not others aligns with the literature on standard drinks indicating that consumers identify greater utility in practice for beer than other beverages [35]. Although within‐plant heterogeneity in % THC makes the applicability of standard units to flower inherently more challenging than other products, this will remain an area in need of clear explanation should standard THC units be implemented. Furthermore, much as all drinkers do not consume the same number of drinks, the concept of a standard THC unit needs clarification that it is not expected that all consumers will use the same number of units and that these will vary according to tolerance, body size and other physiological attributes. The role of these factors and how to incorporate them into a standard THC unit are not well known and should be the target of future research. Educational efforts will be needed to enable even experienced consumers to be well‐versed with the application of a standard metric for cannabis.

### Limitations

The analysis presented above was completed on data derived from adult cannabis consumers for a qualitative study of dosing across cannabis product types. The goal is to generate a deeper understanding of these perspectives and not to generalize to the wider population. The sample primarily included younger adults, and none were older than early 50s. Older adults who use cannabis may engage with the concept of standard THC units in ways that differ from a younger population who may more readily adopt such innovations. Most participants were primarily recreational consumers. Individuals who are strictly medical consumers may have different perspectives. In addition, the interviewees tended to be experienced users of cannabis. Individuals who recently initiated or are soon to initiate into cannabis use may understand standard THC units in distinct ways from those with more experience with cannabis use. Last, we note that standard units only apply to THC content, not to other cannabis compounds.

## CONCLUSION

Although international consensus still must be reached on defining a standard THC unit, such standard metrics may be introduced with enthusiasm from cannabis consumers, whose perceptions largely align with professionals advocating such a metric. Experienced consumers generally believed they would have benefited from a standard THC unit when they first began using cannabis, and they highly recommended it for novices. Key issues to manage for successful implementation include misunderstandings about the concept as well as uptake among regular, experienced consumers. Overall, the adoption of the standard THC unit could be a key strategy for enhanced self‐management of potential acute and long‐term harms associated with cannabis use.

## AUTHOR CONTRIBUTIONS

**Mark Pawson:** Conceptualization; investigation; methodology; writing—review and editing; formal analysis; data curation; project administration. **Mike Vuolo:** Conceptualization; investigation; writing—review and editing; methodology; formal analysis. **Brian C. Kelly:** Conceptualization; investigation; funding acquisition; writing—original draft; methodology; formal analysis; data curation; writing—review and editing.

## DECLARATION OF INTERESTS

M.P., M.V. and B.K. have no financial or non‐financial conflicts of interest to declare.

## Supporting information


**Appendix S1.** Supporting Information.

## Data Availability

Research data are not shared.
