# Supplementary material for: Cannabis consumer responses to the concept of standard THC units
Source: Addiction. 2026 Jun 22;121(10):2747–56. doi: 10.1111/add.70515 (PMC13578826; doi:10.1111/add.70515)
Supplement: Supplementary file 1 [file ADD-121-2747-s001.docx]

**Appendix 1. Description of Standard THC Unit**

The idea of a standard THC unit is along the lines of the concept of a standard drink. People are often familiar that a can of beer is equal to a glass of wine which is equal to a shot of a spirit like vodka or whiskey. We consider all of those things to be “1 standard drink.” There is a similar concept of a standard THC unit to compare across cannabis products. The quantity of THC in cannabis products can be very different depending on what product you buy, especially since some products are sold by percentage THC and others offer milligrams on the package. So, by providing a standard unit, people would be able to make a clear comparison between, for example, a joint of 20% cannabis flower, an 80% vaping pen, and a 10mg gummy by assigning those products a standard THC unit. The gummy may be one standard unit, a joint rolled from the flower 2 standard units, and the entire vaping pen 20 standard units. The number of units depends on the potency of the product, but the standard THC unit makes it easier to compare consumption across product types. People may consume a different number of THC units depending on their size, experience, and desired effects, similar to how different people may consume a different number of drinks when using alcohol.
